# How do the carbon and nitrogen sources affect the synthesis of *β*-(1,3/1,6)-glucan, its structure and the susceptibility of *Candida utilis* yeast cells to immunolabelling with* β*-(1,3)-glucan monoclonal antibodies?

**DOI:** 10.1186/s12934-024-02305-4

**Published:** 2024-01-19

**Authors:** Anna Bzducha-Wróbel, Pavol Farkaš, Sandra Bieliková, Alžbeta Čížová, Marzena Sujkowska-Rybkowska

**Affiliations:** 1 Department of Food Biotechnology and Microbiology, Institute of Food Sciences, Warsaw University of Life Sciences, Nowoursynowska 159C Street, 02-787 Warsaw, Poland; 2 Department of Glycobiotechnology, Institute of Chemistry Slovak Academy of Sciences, Dúbravská Cesta 9, 84538 Bratislava, Slovakia; 3 Department of Glycomaterials, Institute of Chemistry Slovak Academy of Sciences, Dúbravská Cesta 9, 84538 Bratislava, Slovakia; 4 Department of Botany, Warsaw, Institute of Biology, University of Life Sciences, Nowoursynowska 159C Street, 02-787 Warsaw, Poland

**Keywords:** Yeast glucan, Synthesis, Carbon sources, Nitrogen sources, Immunolabelling, Structure analysis

## Abstract

**Background:**

The need to limit antibiotic therapy due to the spreading resistance of pathogenic microorganisms to these medicinal substances stimulates research on new therapeutic agents, including the treatment and prevention of animal diseases. This is one of the goals of the European Green Deal and the Farm-To-Fork strategy. Yeast biomass with an appropriate composition and exposure of cell wall polysaccharides could constitute a functional feed additive in precision animal nutrition, naturally stimulating the immune system to fight infections.

**Results:**

The results of the research carried out in this study showed that the composition of *Candida utilis* ATCC 9950 yeast biomass differed depending on growth medium, considering especially the content of *β*-(1,3/1,6)-glucan, *α*-glucan, and trehalose. The highest *β*-(1,3/1,6)-glucan content was observed after cultivation in deproteinated potato juice water (DPJW) as a nitrogen source and glycerol as a carbon source. Isolation of the polysaccharide from yeast biomass confirmed the highest yield of *β*-(1,3/1,6)-glucan after cultivation in indicated medium. The differences in the susceptibility of *β*-(1,3)-glucan localized in cells to interaction with specific *β*-(1,3)-glucan antibody was noted depending on the culture conditions. The polymer in cells from the DPJW supplemented with glycerol and galactose were labelled with monoclonal antibodies with highest intensity, interestingly being less susceptible to such an interaction after cell multiplication in medium with glycerol as carbon source and yeast extract plus peptone as a nitrogen source.

**Conclusions:**

Obtained results confirmed differences in the structure of the *β*-(1,3/1,6)-glucan polymers considering side-chain length and branching frequency, as well as in quantity of *β*-(1,3)- and *β*-(1,6)-chains, however, no visible relationship was observed between the structural characteristics of the isolated polymers and its susceptibility to immunolabeling in whole cells. Presumably, other outer surface components and molecules can mask, shield, protect, or hide epitopes from antibodies. *β*-(1,3)-Glucan was more intensely recognized by monoclonal antibody in cells with lower trehalose and glycogen content. This suggests the need to cultivate yeast biomass under appropriate conditions to fulfil possible therapeutic functions. However, our in vitro findings should be confirmed in further studies using tissue or animal models.

**Supplementary Information:**

The online version contains supplementary material available at 10.1186/s12934-024-02305-4.

## Background

The complex and dynamic structure of yeast cell wall is mainly built with *β*-(1,3/1,6)-glucan polymer, that forms organized wall matrix via linking with chitin and mannoproteins [1]. The exact structure of the yeast wall still contains some unknowns, inspiring research studies on its composition and organization. It was evidenced in last years that insoluble *α*-(1,4)-glucan (insoluble glycogen) and *β*-(1,4)-linked glucans are also present in the cell wall of *Saccharomyces* yeast, but it is currently not clearly known whether they form cellulose-like structures, occur as mixed-linkages or are interconnected with other cell wall components [2]. In the cell wall matrix of *Candida* species, the presence of covalently linked glycogen has been confirmed recently [3].

Different environmental factors and stresses primarily affect yeast cell walls remodeling which result in different concentration of individual components, but also alter their chemical characteristic and critical yeast cell wall remodeling strongly dependent on host nutritional input [1, 2, 4–6]. There are also species dependent variations in yeast cell wall composition [7]. Cell wall remodeling process is controlled by stress (Hog1) and cell integrity (Mkc1, Cek1) signaling pathways [1]. However, there is not many specific information in literature on how the growth conditions change the chemical characteristic of yeast *β*-(1,3)-glucan or mannoproteins, especially considering non-*Saccharomyces* strains. *β*-(1,3)-Glucan synthase from different fungi, constitutes a rather heterogenous group of enzymes with only a few properties in common. The catalytic subunit of discussed synthase (FKS protein), does not exhibit significant sequence homologies to other glycosyltransferases, form a unique family of GT48, and its catalytic mechanism still remains enigmatic [8–10]. Searching for efficient *β*-(1,3/1,6)-glucan synthesis systems (yeast strains and growth condition) and trying to understand all biosynthesis steps of this polysaccharide, are therefore of current research interests [10, 11]. The implementation of such research is justified by interesting biomedical and technological functions of *β*-(1,3/1,6)-glucan, and other cell wall components, which makes possible their application in different industries, including food and feed production, pharmaceuticals, and cosmetics formulation, or production of new packaging and encapsulants material [12–16]. Yeast cell wall polysaccharides are recognized by different pathogen recognition receptors (PRRs) such as C-type lectins including Dectin-1, -2, and -3, DC-SIGN, mannose-binding receptor, and Toll-like receptors [17]. Yeast origin *β*-(1,3/1,6)-glucan is recognized as pathogen associated molecular pattern via specific *β*-(1,3)-glucan receptors on macrophages. The discussed polysaccharide interacts also with the complement receptor CR3 (CD11b/CD18) and directly activates T and B cells, NK cells, eosinophils, and neutrophils. Structural characteristics of *β*-(1,3/1,6)-glucan, including molecular weight (MW), degree of side-branching, helical conformation, its physical properties, including solubility, and gel-forming ability, possibly affect immune activity of the polysaccharide. *β*-Glucans with higher molecular weight and highly (1,6)-linked were observed to be more stimulatory [17, 18]. Different types of yeast cell wall exerted diverse influences on immunity and disease resistance in fish what could be associated with chemically branched structure of *β*-glucan [19]. Yeast cultivation under optimized conditions seems therefore to be important to obtain biomass with therapeutic properties resulting from the proper composition of cell wall polysaccharides or to develop of specific yeast cell wall fractions towards targeted application, e.g., precision animal nutrition.

The idea to prevent animal diseases rather than cure to minimize the antibiotic resistance of pathogenic microorganisms inspires development of functional feed additives to improve animal health and performance [17]. The search for new therapeutic solutions in animal production, based on natural ways of increasing their resistance to diseases, is consistent with the goals of the European Union Green Deal Strategy and the related Farm to Fork Strategy [20, 21]. According to mentioned strategies, the antibiotic therapies should be limited in animal breeding. At the same time, the reduction of the amount of industrially produced waste is recommended. Healthy, integrated digestive tract is crucial for digestion and absorption of nutrients by animals. Dietary addition of yeast cell wall component may reinforce intestinal integrity and functionality by improving non-specific immune response (phagocytic activity, lysozyme activity), up-regulated immune-related genes expressions and enhanced disease resistance related to improving intestinal health [19].

Agri-food wastes rich in biogenic elements, such as nitrogen or phosphorus, which burdens the natural environment, could be valorized in biotechnological processes in agreement with bio-economy principles. The management of such waste is possible in the cultivation of different microorganisms, including yeast. Yeast biomass could then be used as a feed additive stimulating the natural immune defense of farm animals to fight pathogens infections being a source of proteins and other nutrients at the same time. Deproteinized potato juice water (DPJW) is a waste of the starch industry, rich in nitrogen and microelements. It has been successfully used in yeast cultivation, however it is necessary to enrich DPJW with a carbon source for biomass cultivation to make the process efficient. This can be done using, for example, waste glycerol from biodiesel production or other wastes that are rich in carbon sources [5, 22]. As part of that research, it is worth determining how different, pure carbon sources affect the biosynthesis of yeast cell wall polymers first, especially considering non-conventional species, other than *Saccharomyces*.

The aim of the research was to determine the influence of various carbon and nitrogen sources on synthesis of *β*-(1,3/1,6)-glucan in fodder yeast cells *Candida utilis* ATCC 9950 (syn. *Cyberlindnera jadinii*), immunolabelling of this polymer in cells from applied growth conditions and structural characterization of *β*-glucan isolated from selected biomass samples.

## Methods

### Yeast strain

The yeast strain *Candida utilis* ATCC 9550 was studied which according to the current nomenclature belongs to the *Cyberlindnera jadinii* species. However, we maintain consistency with the name in the ATCC collection. The yeast collected in the Museum of Pure Cultures at Division of Food Biotechnology and Microbiology, Faculty of Food Science, Warsaw University of Life Sciences-SGGW was evaluated.

### Chemicals

Dimethyl sulfoxide and pyridine were purchased from Merck (Germany), iodomethane and sodium borodeuteride from Sigma–Aldrich (USA), sodium hydroxide, chloroform, methanol, and ammonium hydroxide from Slavus (Slovakia), sodium sulfate, formic acid, and acetic anhydride from Lachema (Czech Republic), trifluoroacetic acid from AppliChem (Germany).

### Cultivation media and condition

Yeast cultures were carried out in twelve types of experimental media. Reference (control) cultures were carried out in standard YPG medium with the composition (g/L): 20 g of peptone, 10 g of yeast extract, and 20 g of glucose. The experimental media used nitrogen sources in the form of 1% yeast extract and 2% peptone (YP media) or deproteinized potato juice water (DPJW). The culture media were characterized in terms of total nitrogen content (Kjeldahl method, BÜCHI mineralization and distillation units), directly reducing sugars (only DPJW) determined by the colorimetric method with 3,5-dinitrosalicylic acid (DNS) and selected elements (P, K, Na, Ca, Mg, S), analyzed by the ICP technique in an atomic emission spectrometer (ICP-AES Thermo iCAP 6500 DUO), all according to [5]. The waste deproteinized potato juice water (DPJW) used in the research contained nitrogen in the amount of approx. 2.59 g/L, while the media prepared with the addition of peptone and yeast extract (YP) contained this element in an amount of approximately 4.1 g/L. Small amounts of total sugars (0.93%) were naturally available in DPJW-based media. The media used were a source of minerals in amounts depending on the substrate: YP media contained (amount in mg/L): 8.49; 863; 6.16; 488; 230, and 254, while DPJW contained: 23.7; 5907; 259; 25.1; 419, and 732 of elements: Ca, K, Mg, Na, P, and S, respectively.

The media based on YP and DPJW were supplemented with a carbon source at a concentration of 100 g/L (named lates as 10%). Our previous research showed increased synthesis of *β*-glucan in the presence of 10% glycerol in a medium based on deproteinized potato juice water. The selection of carbon source concentration was also based on the fact, that media containing 10% of each carbon source used guaranteed a more efficient or comparable biomass of the tested yeasts in relation to the YPG control media (considered optimal for yeast growth).

These were the following carbon sources: glycerol, glucose, galactose, maltose. Media containing the simultaneous addition of 100 g/L of glycerol and 15 g/L of glucose were also prepared, and a DPJW media supplemented with 75 g/L of glycerol. This unusual experimental setup regarding the addition of glycerol and its possible combination with glucose resulted from observations made as part of preliminary research. In all media, the initial pH was adjusted to 5.0. The substrates were sterilized at 121 °C; 0.1 MPa for 20 min (HICLAVE HG80 autoclave, Hirayama, Japan).

The YPG medium was also used to multiply the inoculum of the tested yeasts, which were grown in flat-bottom flasks on a shaker operating in the reciprocating mode (200 cycles per minute, SM-30 Control Buechler, Germany) at a temperature of 28 °C for 48 h. Obtained were centrifuged, collected cells suspended in physiological saline, and then inoculated into experimental cultures by adding 10 mL of inoculum to 90 mL of the medium. Experimental yeast cultures were carried out at a temperature of 28 °C for 48 h with reciprocating shaking at a frequency of 200 cycles per minute. At least 5 cultures were carried out in parallel on each experimental medium. After cultivation, the obtained yeast biomass was centrifuged (3500 RPM for 10 min), rinsed twice with distilled water, then freeze-dried (CHRIST LCG GAMMA 1–16 LSC, United Kingdom) and stored in a dried form in tight vessels until analysis. The yield of yeast biomass was determined after 48 h of cultivation in experimental media, expressing the results in grams of dry yeast substance per liter of culture media. The pH was not controlled during the time of cultivation but the culture fluid was analyzed for the post-cultivation pH to determine the direction of changes depending on the carbon source.

### Protein, total sugar, glucans and trehalose analysis in yeast biomass

Freeze-dried yeast biomass from experimental cultures was analyzed for the content of total glucans, *β*-(1,3)/(1,6)-glucan and *α*-glucan using the Mushroom and Yeast beta-glucan Assay Procedure K-YBGL enzymatic test (Megazyme). Based on the glucan content in biomass and the biomass yield, the productivity of *β*-(1,3)/(1,6)-glucan in the experimental cultures was determined and expressed in [g/L]. The content of total sugars was analyzed using the DNS method after prior acid hydrolysis of the biomass using 72% sulfuric acid. Nitrogen was determined using the Kjeldahl method (approx. 5-h mineralization of 50 mg of sample in the KjelFlex K-360 (Buchi) with the presence of 96% sulfuric acid and the Kjeltabs CT/3.5 catalyst. After distilling off the nitrogen, automatic titration of the samples was carried out using the TitroLine 5000 device (SI Analytics) and standard 0.1 M HCl solution. Nitrogen was converted to protein using the factor 6.25. The trehalose content was determined using the Trehalose Assay Procedure K-TREH enzyme test, Megazyme. The biomass (20 mg) was lysed in the presence of 0.5 mL of Y-PER reagent (Thermo Scientific) to release trehalose. The samples were incubated for 1 h at room temperature, while vortexing the samples several times. After incubation, the samples were centrifuged for 3 min at 15,000 RPM (MiniSpin Plus EPPENDORF). The supernatant was then collected in an appropriate amount to perform trehalose determinations in accordance with the test manufacturer's instructions.

### Immunolabeling of *β*-(1,3)-glucan and transmission electron microscopy observations

Immunolocalization of *β*-(1,3)-glucan at the TEM level was carried out after yeast cells were fixed in 4% paraformaldehyde with the addition of 2% glutaraldehyde based on 0.01 M PBS buffer (pH 7.4), dehydrated in increasing concentrations of ethyl alcohol and embedded in epoxy resin. Ultrathin sections were transferred to nickel grids with formvar and immunolabeled. Grids were treated with blocking buffer containing 3% BSA in 0.01 M PBS buffer (pH 7.4), then incubated with a primary *β*-(1,3)-glucan specific antibody (mouse anti-1,3 beta-glucan [2G8] Abcam), then with secondary antibody conjugated with 10 nm colloidal gold (goat anti-mouse IgG (whole molecule)–Gold antibody, SIGMA-ALDRICH). The prepared grids were observed using TEM (FEI 268D Morgagni, as well as JEM-1220 type JOEL).

### Glucan isolation

The *β*-(1,3/1,6)-glucan preparations were isolated from freeze-dried biomass using extraction with 0.1 M NaOH solution at first step. Samples were incubated in a water bath at 60 °C for 30 min. Then the extraction was repeated at 115 °C for 45 min. The next step was to rinse the sediment with distilled water while after, they were suspended in 0.1 M acetic acid. The samples were incubated at 85 °C water bath for 1 h. Then, 3% H_2_O_2_ solution was added to the sediments. The samples were mixed thoroughly and left at a temperature of 4 °C for 3 h. The next stages of the procedure were rinsing the sediments with distilled water and sonication in an ultrasonic bath for 12 min. During the next step samples were incubated in a 1% NaClO solution. Then, the white glucan sediments were suspended in distilled water and washed. Obtained pellets were suspended in 96% ethanol and incubated for min. 3 h. Subsequently, the sediments were washed several times with water and freeze-dried. Lyophilized samples were stored in this form until NMR analysis.

### Elemental analysis of isolated glucan preparations

Elemental analysis of isolated glucan preparations was performed using FLASH 2000 Organic elemental analyzer (CHNS-O), Thermo Fisher Scientific. Temperature: Left furmance: 950 °C. Oven (separation column PTFE; 2 m; 6 mm × 5 mm): 65 °C. Gas chromatographic column material: Steel, length: 2 m, diameter: 6 mm × 5 mm. Flow: Carrier gas: He: 140 mL min^–1^. Reference gas He: 100 mL min^–1^. Gas for sample oxidation: O2, 250 mL min^–1^. Minimum purity of He, 99.995%. System timing: Cycle (run time: 720 s. Sampling delay: 12 s. Oxygen injection end: 5 s. Filament: TCD detector: Instrument control: Eager Xperience for Windows. Analytical scales: Mettler Toledo XP6.

### FITR-ATR of isolated glucan preparations

FT-IR spectra were measured on Nicolet™ iS50 FT-IR spectrometer (Thermo Fisher Scientific) equipped with DTGS detector, and OMNIC 9.0 software. Infrared spectral analyses were carried out in mid-infrared region (from 4000 to 400 cm^−1^) at a resolution of 4 cm^−1^ and spectral data obtained were presented as absorbance values. The number of scans was set to 32. Diamond Smart Orbit ATR accessory was used for measurements in solid state. For ATR-FTIR spectra acquisition, a finely powdered sample was applied onto the diamond window of the ATR device and measured. Spectra were recorded, referencing a background taken in the air before each spectrum, and under identical measurement conditions.

### NMR analysis of isolated *β*-glucan preparations

Glucan preparations isolated from samples selected based on the results of biosynthesis yield and efficiency of immunolabelling were characterized structurally by ^1^H NMR spectra which were acquired in 2.5 mass % LiCl/DMSO-d6 (99.80% D) at 65 °C on a Bruker AVANCE III HD 400 MHz (Bruker BioSpin, Rheinstetten, Germany) with broad band BB-(H-F)-D-05-Z liquid N2 Prodigy probe and processed using TopSpin 4.1.4 software (Bruker). The ^1^H signal of DMSO (2.5 ppm) was used as a reference for chemical shifts. Glucan samples (10 mg) were dissolved in 600 μL of 2.5 mass % LiCl in DMSO-d6 (99.80% D) at 105 °C for 1 h with the addition of 10 μL TFA just prior to analysis. The solubility of glucans in 2.5 mass % LiCl/DMSO-d6 differed. In some cases, highly viscose or opaque gel was formed. An extra volume of DMSO-d6 was added in order to load enough sample into the cuvette. Similarly, an extra volume of TFA was necessary to shift the exchangeable proton resonances downfield. The side-chain length (SC) and branching frequency (BF) were calculated according to [23] using the following formulae: SC = H6SC / (H6SC – H1SC) (1), BF = H1BB / (H6SC – H1SC) (2), where H1SC is an integrated area of resonances assigned to H1 of the side chain (4.25 ppm); H6SC is an integrated area of resonances assigned to H6 of the side chain (3.97 ppm), and H1BB is an integrated area of resonances assigned to H1 of the backbone (4.49 ppm).

The relative content of glycogen in glucan samples was calculated according to [3] using the following formula: Glycogen content = H1gly* / (H1gly* + H1SC + H1BB) (3) where H1SC is an integrated area of resonances assigned to H1 of the side chain (4.25 ppm); H1BB is an integrated area of resonances assigned to H1 of the backbone (4.49 ppm); H1gly* is an integrated area of resonances assigned to H1 of the glycogen (4.96–5.36 ppm). From spectra of the glucans, it is apparent, that the main portion of the glycogen is 1,4-linked Glc, there is no or hardly a trace of the peaks of 1,6-linked Glc (around 4.7 ppm).

### Linkage analysis

Partially methylated alditol acetates (PMAA) were prepared according to [24], with slight improvements. Briefly, glucan samples were dissolved and methylated. Methylation efficiency was controlled by FT-IR, with the absence of the broad absorption band in 3200–3700 cm^–1^ (OH stretching). FT-IR analyses were held on Nicolet™ iS50 FT-IR spectrometer (Thermo Fisher Scientific) and spectra were viewed in OMNIC™ software (Thermo Fisher Scientific). Next, the permethylated samples were hydrolyzed with formic acid and trifluoroacetic acid, reduced with sodium borodeuteride and peracetylated with pyridine and acetic anhydride solution. Gas chromatography-mass spectrometry (GC–MS) was held on Trace GC Ultra, TSQ Quantum XLS (Thermo Fisher Scientific) with SP-2330 column and conditions: 80 °C (4 min); 160 °C (8 °C/min, 4 min at 160 °C); 250 °C (4 °C/min 250 °C, 12 min at 250 °C). GC chromatograms and MS spectra were analyzed in XcaliburTM Software (Thermo Fisher Scientific).

### Statistical analysis

The obtained results were subjected to statistical analysis using the STATISTICA V.13.1 program. A one-way analysis of variance was performed using the NIR test with a significance level of *α* = 0.05 to define the importance of noticed differences.

## Results and discussion

### Biomass yield and chemical composition

The culture media used stimulated the growth of the cells of the tested yeast *C*. *utilis* ATCC 9950 to varying degrees (Table 1). The most favorable conditions for the tested yeasts were found in media based on deproteinized potato juice water (DPJW) as a nitrogen source, supplemented with glycerol, maltose and glucose as a carbon source. The biomass yield obtained in these substrates after 48 h of cultivation was approximately 24–29 g/L, depending on the medium. The type of nitrogen source significantly influenced the biomass efficiency, because in substrates containing yeast extract and peptone, and the same carbon source as in DPJW, the biomass yield was significantly lower (between approx. 10 g/L and 17.5 g/L). In the YPG control medium containing 2% glucose, the yield was approximately 10 g/L.

**Table 1:** The biomass yield and composition after 48 h of cultivation of *Candida utilis* ATCC 9950 in different growth media and pH of the media after 48 h of cultivation

Growth medium	Biomass yield/(g/L)	Protein/%	Total sugars/%	pH
YPG	10.5 ± 2.2 a*	32.3 ± 0.4 b,c	40.0 ± 1.9 a,b	7.72
YP_Glc 10%	13.6 ± 1.0 a,b	32.0 ± 0.1 a,b,c	46.0 ± 1.9 d	4.71
DPJW_Glc 10%	24.0 ± 1.1 d	33.2 ± 0.5 c	39.4 ± 1.3 a	4.79
YP_Glyc 10%	16.4 ± 4.2 b,c	30.1 ± 1.0 d	42.2 ± 2.0 b,c	6.57
DPJW_Glyc 7.5%	24.5 ± 2.3 d,e	30.9 ± 0.8 a,b,d	39.6 ± 1.2 a	7.34
DPJW_Glyc 10%	25.9 ± 3.2 d,e	30.7 ± 0.2 a,d	40.5 ± 1.8 a,b	7.40
YP_Glyc 10% + Glc 1.5%	18.4 ± 2.2 c	30.0 ± 0.2 d	46.7 ± 1.8 d	6.02
DPJW_Glyc 10% + Glc 1.5%	23.5 ± 4.3 d,e	31.7 ± 0.4 a,b,c	41.5 ± 3.2 a,b,c	7.18
YP_Gal 10%	10.4 ± 2.3 a	32.2 ± 0.4 a,b,c	43.1 ± 2.2 c	6.47
DPJW_Gal 10%	17.6 ± 2.1 b,c	32.1 ± 1.7 a,b,c	40.7 ± 2.6 a,b	5.91
YP_Malt 10%	17.5 ± 2.6 b,c	31.1 ± 0.5 a,b,d	41.7 ± 3.0 a,b,c	4.70
DPJW_Malt 10%	28.6 ± 5.4 e	32.2 ± 1.1 a,b,c	42.4 ± 2.3 b,c	5.42

*YPG* yeast extract + pepton + 2% glucose medium, *YP* yeast extract + peptone, *DPJW* deproteinated potato juice water, *Glyc* glycerol, *Glc* glucose, *Gal* galactose, Malt – maltose; * a, b, c, … mean values in column marked with the same letters do not differ significantly

The final pH of the culture depended primarily on the type of carbon source—the direction of changes was similar in the presence of various nitrogen sources. Yeast multiplication in the presence of glucose at the concentration of 10% (w/v) and in the presence of maltose (the exception was the medium with maltose and DPJW), caused a slight acidification of the environment compared to the initial pH of the medium (pH 5.0), while the metabolism of glycerol and galactose contributed to deacidification and alkalization of the medium. This process also took place during cultivation in a control medium with significantly lower glucose availability. Glucose, maltose, and galactose promoted protein synthesis in cells, while glycerol limited this process. In the case of sugars in biomass, the trend was not so obvious—their synthesis depended on the type of nitrogen source in the medium.

The results obtained confirmed previous observations regarding the tendency of the *C*. *utilis* ATCC 9950 strain to synthesize significantly higher amounts of *β*-(1,3/1,6)-glucan in the DPJW medium with the addition of 10% glycerol, but also when it was added at the concentration of 7.5% [5, 22]. The content of discussed cell wall polysaccharide in the biomass of the tested yeasts from the indicated growth conditions was: 27.1 g and 28.4 g per 100 g of biomass, respectively, with approx. 23.5 g per 100 g after multiplication in YPG control medium (Table 2). In the YP medium with glycerol as the only carbon source, reduction in the content of the *β*-(1,3/1,6)-glucan (to approx. 20.5 g per 100 g of biomass) was observed. While glycerol and glucose were available in the YP medium, the concentration of the polysaccharide in biomass was comparable to that after cultivation in control medium. According to data analysis presented by [25], when the crude membranes were used as source of yeast *β*-(1,3)-glucan synthetase, the maximal catalytic activity was obtained at pH 8.0 in the presence of UDP-glucose as substrate, GTP, glycerol, and bovine serum albumin. However, in the presence of both glycerol and glucose in the DPJW medium, our observation was opposite. The synthesis of *β*-glucan was limited to a level of approximately 23.9 g per 100 g of biomass compared with medium supplemented only with glycerol. This is an interesting observation. Presumably, the level of sugars naturally present in DPJW (approx. 0.63% as indicated in the methodology) in combination with glucose influenced the observed tendency of cells to limit the synthesis of *β*-glucan in the presence of glycerol comparing with medium only with glycerol. In previous research we observed a similar relationship when the natural sugar content in DPJW was approximately 1.5–2.0% and the addition of glycerol was 10%.

**Table 2:** The content of total-, *α*-, *β*-glucans and trehalose in the dry biomass of studied yeast after cultivation in different growth media

Sample	Total glucans [g/100 g dry biomass]	*α*-glucan [g/100 g biomass]	*β*-(1,3/1,6)-glucan [g/100 g biomass]	*β*-(1,3/1,6)-glucan yield [g/L culture]	Trehalose [g/100 g biomass]
YPG	24.50 ± 0.94 a,b*	0.98 ± 0.09 a	23.52 ± 1.03 b	2.48 ± 0.55 a	3.77 ± 0.96 e,f
YP_Glc 10%	29.22 ± 1.12 c	5.19 ± 0.06 c	24.03 ± 1.06 b	3.27 ± 0.19 a,b	5.34 ± 1.79 g
DPJW_Glc 10%	29.49 ± 0.16 c	8.96 ± 0.50 e	20.52 ± 0.66 a	4.77 ± 0.17 d,e	5.50 ± 0.75 g
YP_Glyc 10%	23.17 ± 0.42 a	0.95 ± 0.19 a	22.21 ± 0.61 a,b	3.64 ± 0.95 b,c	1.78 ± 0.70 b,c
DPJW_Glyc 7,5%	29.41 ± 0.92 c	1.05 ± 0.28 a	28.36 ± 1.20 d	6.98 ± 0.54 g	1.90 ± 0.33 b,c
DPJW_Glyc 10%	28.32 ± 1.34 c	1.23 ± 0.01 a,b	27.10 ± 1.33 c,d	7.03 ± 0.95 g	1.34 ± 0.56 b
YP_Glyc 10% + Glc 1.5%	23.98 ± 2.34 a,b	0.92 ± 0.02 a	23.21 ± 2.64 b	4.60 ± 0.21 c,d,e	2.97 ± 0.31 d
DPJW_Glyc 10% + Glc 1.5%	25.17 ± 0.66 a,b	1.22 ± 0.01 a,b	23.94 ± 0.65 b	5.62 ± 1.08 e,f	1.58 ± 0.52 b
YP_Gal 10%	26.06 ± 1.60 b	1.32 ± 0.02 a,b	24.74 ± 1.58 b,c	2.57 ± 0.60 a	3.03 ± 0.90 d,e
DPJW_Gal 10%	25.94 ± 0.20 b	1.76 ± 0.03 b	24.18 ± 0.16 b,c	4.21 ± 0.49 b,c,d	0.45 ± 0.19 a
YP_Malt 10%	29.75 ± 1.47 c	7.27 ± 0.56 d	22.49 ± 0.91 a,b	3.91 ± 0.65 b,c,d	4.48 ± 1.04 f
DPJW_Malt 10%	32.72 ± 0.69 d	10.20 ± 0.31 f	22.51 ± 0.38 a,b	6.43 ± 1.15 f,g	2.48 ± 0.32 c,d

*YPG* yeast extract + pepton + 2% glucose medium, *YP* yeast extract + peptone, *DPJW* deproteinated potato juice water, *Glyc* glycerol, *Glc* glucose, *Gal* galactose, Malt – maltose; * a, b, c, … mean values in column marked with the same letters do not differ significantly

The highest yields of *β*-(1,3/1,6)-glucan were achieved in DPJW media containing 7.5% and 10% glycerol (approx. 7 g/L). The high yield of *β*-(1,3/1,6)-glucan was also observed after cultivation in a presence of maltose and DPJW, but it was because the biggest biomass yield achieved under discussed growth condition. In the biomass from the indicated culture, as well as in samples collected from other media with glycerol, regardless of the nitrogen source, significantly lower contents of trehalose and *α*-glucan were typical, which confirms that in the presence of glycerol, *β*-glucan was the favored metabolic product. In the presence of maltose and glucose at the concentration of 10%, cells intensively synthesized both *α*-glucan and trehalose. In the case of a DPJW substrate with the addition of glucose, this resulted in a significant reduction in the *β*-glucan content in the biomass (up to approx. 20.5%) comparing with YPG medium. Galactose did not induce a response in cells that would be demonstrated by increased synthesis of the saccharides discussed. After multiplication in the YP medium, the content was comparable to resulted in the biomass from the YPG control medium.

The last results presented by [26] evidenced that peptide supplementation may positively modulate the CWI pathway and up-regulate the expressions of several genes associated with *Saccharomyces* cell wall remodeling. The mentioned supplementation increased the thickness of cell wall via higher levels of chitin, mannan, and *β*-glucan in the wall structure. Decreased degradation of cell wall proteins under osmotic stress was also observed. Thus, the composition of nitrogen source may modulate the structure of yeast cell wall. Considering above, the peptide composition of DPJW and YP should be examined in the future to describe the differences between mentioned waste and the medium based on yeast extract and peptone.

Glycogen is an *α*-(1,4)-linked polymer synthesized by glycogen synthase from uridine-diphospho-glucose (UDP-Glc) as a glucose donor. It is a highly branched polymer with branch points via *α*-(1,6)-glycosidic linkages. The polysaccharide acts as storage material of both carbon and energy under starvation. The glycogen content of yeast differs depending on growth conditions. Yeast cells, when grown in media with higher concentration of sugar or when subjected to nutritional stress conditions, can store higher levels of glycogen and its content in total yeast dry cell weight ranges from 1 to 23% [27]. With the degree of branching the glycogen solubility increases, as well as the number of non-reducing ends of *α*-(1–4)-linked chains at which the polymer can be both broken down and re-synthesized. It was noted that increased glycogen synthesis in yeast results in less branched polymer. The branching rate is also influenced by composition of growth media, being more branched after cultivation in optimal medium (YPG) compared with limited one [28]. The variations in the level of soluble and insoluble yeast glycogen were observed after cultivation in media with various sugar concentrations. The glycogen increased exponentially with increasing sugar concentration up to 10% [27]. The concentration of 10% sugar was proposed in quoted work as optimum for maximum growth and deposition of glycogen in yeast because at higher concentrations of sugars catabolic substrate repression was observed. It was suggested that membrane-bound insoluble glycogen might play a protective role because its deposition was observed under ethanol stress.

Trehalose is highly stable, non-reducing disaccharide, *α*-D-glucopyranosyl-(1 → 1)-*α*-D-glucopyranoside. It is known as a reserve carbohydrate and protectant of proteins and biological membranes against a variety of environmental and nutritional stresses. The biosynthesis of trehalose includes the UDP-glucose transfer to glucose-6-phosphate (G6P) by trehalose-6-phosphate synthase to produce trehalose-6-phosphate. The last is dephosphorylated by trehalose-6-phosphate phosphatase to trehalose. Biosynthesis of discussed disaccharide is negatively regulated by free inorganic phosphate (Pi). Trehalose biosynthesis is required for the regulation of cell wall homeostasis in different fungi [29, 30]. It can accumulate up to 15% of the cell dry mass in *Saccharomyces cerevisiae* cells, depending on the growth conditions, the stage of the life cycle, or environmental stress [29].

All trehalose-6-phosphate synthase, glycogen synthase and *β*-(1,3)-glucan synthase compete for the same substrate, UDP-glucose [31, 32]. It was proposed that diversion of UDP-glucose into trehalose may reduce a glycogen synthase substrate and reduction of glucose-6-phosphate eliminates a glycogen synthase allosteric activator [31].

### Immunolabeling of *β*-(1,3)-glucan in studied cells from different growth media

**Fig. 1:**
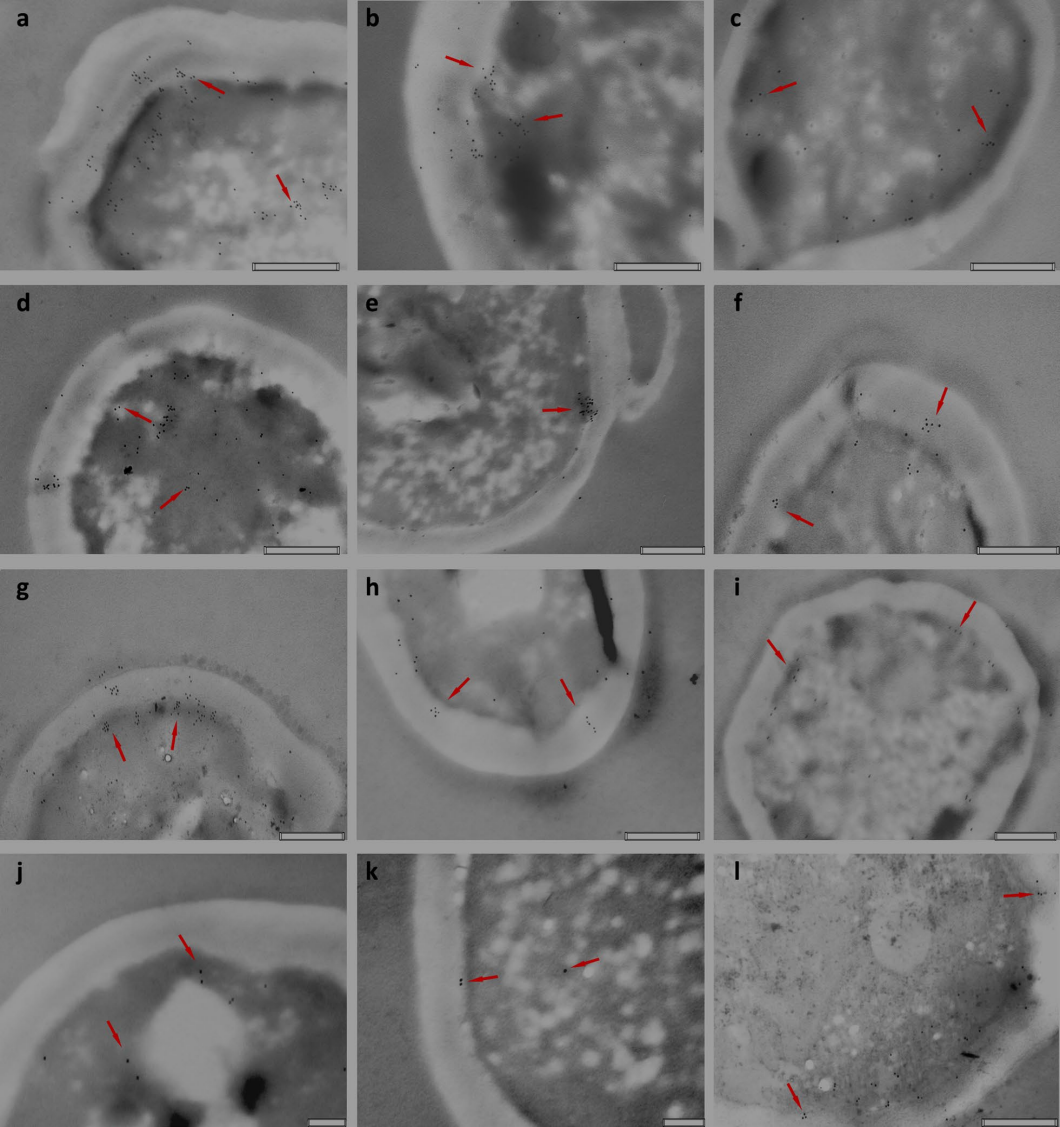
Transmission electron microscopy visualization of *β*-(1,3)-glucan immunocytochemically labeled using anti-*β*-(1–3)-glucan antibodies in *C*. *utilis* ATCC 9950 cells depending on the culture conditions: **a** DPJW_Glyc 7.5%; **b** DPJW_Glyc 10%; **c** DPJW_Glyc 10% + Glc 1.5%; **d** YP_Gal 10%; **e** YP_Glyc 10%; **f** YP_ Glyc 10% + Glc 1.5%; **g** DPJW_Gal 10%; **h** YP_Malt 10%; **i** DPJW_Malt 10%; **j** YP_Glc 10%; **k** DPJW_Glc 10%; **l** YPG. Arrows indicate gold-labeled glucan. Bars = 500 nm

The analysis of the ultrastructure of the tested yeast cells from experimental cultures using TEM showed the presence of irregular clusters of the substance accumulated in the cytoplasm and within the cell wall, similarly to our previous observations described so far only for cells from DPJW medium with the addition of glycerol [22]. Such clusters were also present in cells after cultivation in the presence of maltose, galactose, and glucose from the experimental model media discussed in current work, although their intensity seemed to depend on the type of carbon and nitrogen source. The most intense ones were observed in cells propagated in the presence of glycerol, maltose, and galactose added to DPJW. Their nature resembles the material from which the cell wall is made in the inner layer, i.e., the glucan layer. This material in the boundary layer seemed to be incorporated into the wall structure, which is most clearly documented in photos a, b, d, and g (Fig. 1). Immunocytochemical labeling using antibodies reacting specifically with the ligands present in *β*-(1,3)-glucan molecules (anti-*β*-(1–3)-glucan, Abcam) and antibodies labeled with colloidal gold showed the presence of the indicated polysaccharide in the yeast wall and in the cytoplasm of cells in the boundary layer (Fig. 1). Interestingly, in the case of cells propagated in DPJW with the addition of glycerol, *β*-(1,3)-glucan antibodies bind intensively in clusters also in the deeper layers of the cytoplasm, localizing the polysaccharide also in in these places of the cell (Fig. 1a, b). The most intense immunolabeling was observed in yeast biomass from cultures containing glycerol and galactose as a carbon source and DPJW as a nitrogen source. It is surprising that in cells from the control medium and others containing glucose and maltose regardless of the nitrogen source, glucan immunolabeling was less intense. According to producer information, the anti-*β*-(1,3)-glucan antibody used (ab233743, Abcam) reacts specifically with their respective oligosaccharide fragments in which the glucoses are connected by *β*-(1,3)-glycosidic bonds, and occasionally with fragments containing chain branching through single glucosyl residues connected by a *β*-(1,6)-glycosidic bond. The ab233743 antibody has been shown to be effective in multiple models of fungal diseases including vaginal and systemic *Candida* infection, invasive *Aspergillosis*, and *Cryptococcus neoformans* infections. The intensity of immunolabeling, related to the specificity of recognition and binding strength of ligands by the antibody, may results from the structural differences and conformation of yeast *β*-(1,3/1,6)-glucan, which change under the influence of the growth conditions of these microorganisms. A polymer with a longer linear chain and less branching of the molecule may contain more antibody binding sites. The obtained results suggest that the distribution of the binding sites of the antibody used changed depending on the growth conditions of the tested yeast. The antibody could also require the presence of oligosaccharides of a certain length, which may be related to the stage of *β*-glucan synthesis. At the same time, in the tested yeast cells, other cellular components could influence the interaction of *β*-(1,3)-glucan with the antibody. It is noticeable, for example, that less intense immunolabeling under the culture conditions indicated above, was observed in cells in which *α*-glucan and/or trehalose were present in the largest amounts (Table 2). In cells from glycerol and DPJW media, these components were present in the smallest amounts, and immunolabeling was the most intense. The presence of *α*-glucan in the wall structure of the tested yeast is confirmed by the results of structural studies discussed below. The pathogenic yeast adopts strategies to evade immune recognition by masking cell wall *β*-(1,3)-glucan molecules. For example, mannoproteins present in cell wall structure could affect the *β*-(1,3)-glucan exposition to immunolabeling. This mannosylated glycoproteins are connected to the inner *β*-(1,3)-glucan layer and chitin by modified GPI anchors and *β*-(1,6)-glucan linkages and prevent availability for immunogenic system/receptors/molecules [33].

According to the data on *β*-(1,3/1,6)-glucan synthesis in *Saccharomyces cerevisiae* yeast the process is based on the initiation, the elongation of glucan chains and the branching steps. The reaction of *β*-(1,3)-glucan chain elongation is well known only. The enzymatic glucan synthase complex consists of a catalytic subunit (FKS1 and FKS2), with probable overlapping functions, and a regulatory RHO subunit – G-protein which activates FKS through dephosphorylation of guanosine triphosphate (GTP). The RHO activity is modulated by ROM, a wall GDP-GTP exchange factor protein, under environmental stress that causes alterations in the yeast cell wall structure. The cytoplasmic uridine diphosphate glucose (UDPG) is used as the glycosyl donor by plasma membrane-associated protein complex (GLS complex) placed towards the cytoplasm and the cytosolic domains of FKS subunit catalyzes the homopolymerization reaction in the cytosol [9, 32]. It was reported recently [9] that yeast glucan synthase produces in vitro* β*-(1,3)-glucan with the degree of polymerization (DP) of 6550 ± 760, what means comparable to the DP of *β*-(1,3)-glucan in yeast cell walls. Previous reports suggested the DP app. 60–80. These analyses suggest that the enzyme catalyzes formation of multiple glucan chains, releases the *β*-(1,3)-glucan products and re-initiates polymerization of a new glucan chain. The linear polymer is then transferred to the periplasmic space by a transmembrane enzyme complex and incorporated into the fungal cell wall. The *β*-(1,6)-glycosidic side branches are formed there, which are important for linking several *β*-(1,3)-glucan chains together to form cell wall matrix in a multi-step process. *β*-(1,6)-glucan is not detectable intracellularly. Considering the above, the immunolocalization of *β*-glucan in the cytoplasm within the cell wall, observed in our studies, is understandable. It is clearly visible that material from these regions is incorporated into the wall structure. On the outside of the wall *β*-(1,3)-glucan chains are cross-linked to chitin by Crh1 and Crh2 chitinglucanosyltransferases, elongated into full length glucan by Gas1 family *β*-(1,3)-glucanosyltransferases and PIR-like proteins are attached. In this area, the glucan may already be masked by other wall components, what makes the interaction with antibodies difficult. The FKS1 is expressed during cultivation under optimum growth conditions, while FKS2 is induced under environmental stress, e.g., glucose limitation, growth on acetate, glycerol, or galactose; when cells are treated with Ca^2+^ or stressed by shift to high temperature [9, 11, 32]. It was indicated that FKS transits the secretory pathway because in vesicular transport mutants it was accumulated intracellularly, and its activity was sensitive to phytosphingosine levels in the ER [32]. Perhaps, the limited activity of trehalose and glycogen synthesis causes higher availability of UDP-glucose in the presence of glycerol. Glycerol as a non-fermentable alternative carbon source is utilized via gluconeogenesis pathway [34]. This could contribute to increased activity of *β*-glucan synthase, perhaps also during the transit from the deeper parts of the cytoplasm.

It was noted that trehalose‐6‐phosphate synthase (TPS) plays an important role in regulating carbohydrate metabolism, growth and development, and response to stress.

It affects the cellular polysaccharides synthesis, like synthesis of lipopolysaccharides (LPS), extracellular polysaccharides (EPS), and causes changes in cell wall structural integrity and its polysaccharide content. The effect of trehalose on cell wall composition has been evidenced both in plants and fungi. For example, blocking the trehalose synthesis pathway affected cell wall homeostasis in *Aspergillus fumigatus*. It was proposed that the TPS1 protein rather than trehalose accumulation in yeast cells plays an important role in protecting cells against abiotic stresses [35]. The intermediate of trehalose biosynthesis, trehalose-6-phosphate, was identified as an important regulator of yeast sugar metabolism and signaling. Its synthesis controls yeast gluconeogenesis downstream and independent of SNF1. Activation of gluconeogenesis occurs when cells are starved for glucose and require a functional SNF1. The SNF1 kinase complex is required for the induction (derepression) of enzymes involved in gluconeogenesis, respiration, uptake and metabolism of alternative carbon sources, such as glycerol, ethanol, and sucrose, and a selection of glucose transporters [36].

### Structural characterization of *β*-glucan preparation isolated from selected biomass samples

**Fig. 2:**
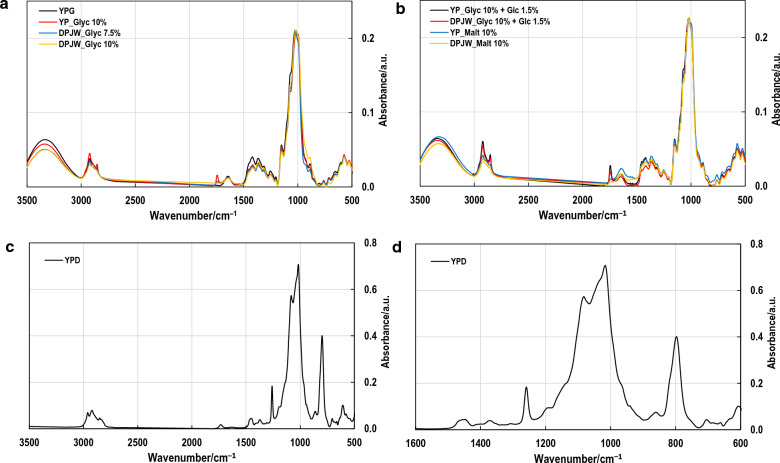
FT-IR spectra of glucans from selected biomass samples: YPG, YP_Glyc 10%, DPJW_Glyc 7.5%, DPJW_Glyc 10% (**A**); YP_Glyc 10% + Glc 1.5%, DPJW_Glyc 10% + Glc 1.5%, YP_Malt 10%, DPJW_Malt 10% (**B**); permethylated *β*-glucan sample isolated from the biomass cultivated in control YPG medium (**C**); and detail of anomeric region of per-methylated sample (**D**)

**Fig. 3:**
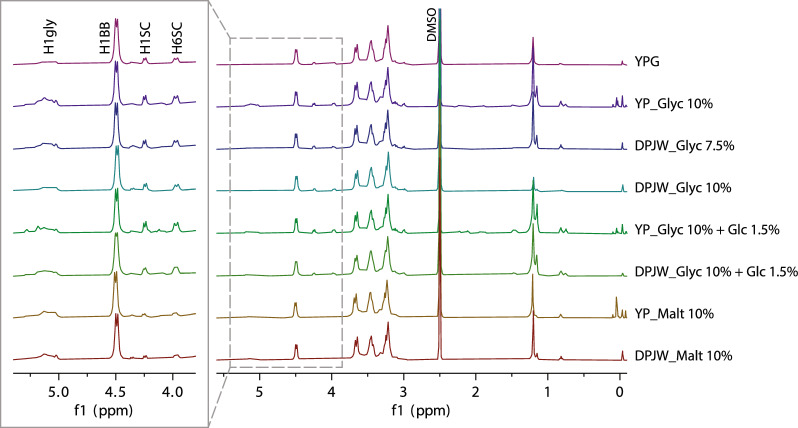
^1^H NMR spectra of the obtained glucan preparations. The spectra were acquired in 2.5 mass % LiCl/DMSO-d6 (99.80% D) at 65 °C. The ^1^H signal of DMSO (2.5 ppm) was used as a reference for chemical shifts. The extension of spectral region from 5.4 to 3.8 ppm shows the resonances of the H1 protons of glycogen (H1gly), *β*-(1,3)-glucan backbone (H1BB), and H1 (H1SC) and H6 (H6SC) protons of *β*-(1,6)-glucan side chains

To determine potential differences in the structure of the *β*-(1,3/1,6)-glucan polymer depending on the culture medium in which the tested yeast cells of *Candida utilis* ATCC 9950 were grown, the structural analysis of this polymer was performed. It was based on FITR-ATR (Fig. 2A, B) and ^1^H NMR spectra analysis (Fig. 3) and linkage analysis by partially methylated alditol acetates (PMAA) examination. This stage of research was performed for glucan extracted from selected biomass samples, based on the high content of *β*-glucan in cells and its yield, as well as *α*-glucan and trehalose content.

The isolation procedure used allowed obtaining *β*-glucan preparations that were not contaminated with protein (Table 3). The highest yield (approx. 8.5%) of the preparation was achieved when the biomass grown in the DPJW medium with 10% glycerol was used for isolation. This is in agreement with the results obtained from the biomass analysis (Table 1). The FTIR-ATR spectra were typical for yeast *β*-glucan (Additional file 1: Table S1).

**Table 3:** Elemental analysis results of isolated *β*-glucan preparations and yield of the preparation isolated from selected yeast biomass

Sample	*β*-Glucan Mass fraction/%			Yield/%
	Nitrogen	Carbon	Hydrogen	
YPG	0.00	41.94	6.68	5.4 ± 0.7 b
YP_Glyc 10%	0.00	43.78	7.00	4.5 ± 0.3 a,b
DPJW_Glyc 7.5%	0.00	42.99	6.98	6.2 ± 0.9 b
DPJW_Glyc 10%	0.00	41.05	6.52	8.5 ± 0.5 c
YP_Glyc 10% + Glc 1.5%	0.00	45.60	7.20	6.0 ± 1.3 b
DPJW_Glyc 10% + Glc 1.5%	0.00	44.80	7.22	6.0 ± 0.2 b
YP_Malt 10%	0.00	41.98	6.65	4.0 ± 0.4 a
DPJW_Malt 10%	0.00	42.27	6.60	5.1 ± 0.5 b

*YPG* yeast extract + pepton + 2% glucose medium, *YP* yeast extract + peptone, *DPJW* deproteinated potato juice water, *Glyc* glycerol, *Glc* glucose, *Gal* galactose, *Malt* maltose

The linkage analysis via preparation of partially methylated alditol acetates (PMAA) is a powerful tool widely used in chemistry of polysaccharides [37]. Results of gas chromatography separation and mass spectra (MS) were analysed and compared with our PMAA standards, retention times and literature focused on PMAA ion fragmentation rules and patterns [37–39]. Since the FT-IR spectra of methylated samples were nearly identical, the representative spectrum for glucan isolated from control medium was shown (Fig. 2C, D). The absence of OH stretching in 3200–3700 cm^–1^ confirms the methylation efficacy. The most important spectral regions for structural characterization of polysaccharides are the „sugar region” (1200–950 cm^–1^) with overlapping C–O and C–C stretching of glycosidic bonds and pyranoid ring, peaks around 2920 cm^–1^ (C–H stretching) and anomeric region (950–750 cm^–1^) with weak bands assigned to anomeric structure [40–43]. Analysis of PMAA resulted in clear identification of certain D-glucopyranosyl (Glc*p*) derivatives and corresponding linkage types (Table 4). The results showed one linkage pattern typical for all glucan samples. Major part of Glc*p* residues matches to *β*-(1,3/1,6)-glucan, typical yeast cell wall glucan, composed of 1,3-backbone glucopyranose strains and 1,6-linked branches. The huge portion of 2,3,6-Me_3_ Glc*p* indicates the co-isolation of *α*-(1,4)-glucan, also known as yeast glycogen, which is difficult to remove in purification processes [7]. This explanation is supported with the FT-IR analysis, with the bands in anomeric region corresponding with *α*- (870–840 cm^–1^) and *β*- (890–925 cm^–1^) conformers (Fig. 2D) [40]. Yeast glycogen serves as a glucose store and its content adapts in response to different environmental changes. Its metabolism is controlled by complex regulatory systems [44]. However, two pools of glycogen are recognized in *Saccharomyces* origin cells. In cytosol the soluble glycogen is located, while the insoluble form of glycogen is covalently linked to the cell wall *β*-(1,3)-glucans through a *β*-(1,6)-linkage. Another pool of glycogen important for flocculation was identified on the cell surface of bottom fermenting yeast [14]. It was evidenced recently that glycogen is linked to *β*-(1,3/1,6)-glucan via the *β*-(1,6)-linked side chain in yeast cell wall of different *Candida* species, forming macromolecular complex in the cell wall [3].

**Table 4:** Linkage analysis of *C*. *utilis* glucans. Values represent relative mole %

Sample	Glc*p* derivative							
	2,3,4,6-Me_4_	2,4,6-Me_3_	2,3,6-Me_3_	2,3,4-Me_3_	2,6-Me_2_	4,6-Me_2_	2,4-Me_2_	2,3-Me_2_
	Type of linkage							
	Glc*p*-(1 →	→ 3)-Glc*p*- (1 →	→ 4)-Glc*p*- (1 →	→ 6)-Glc*p*- (1 →	→ 3,4)-Glc*p*- (1 →	→ 2,3)-Glc*p*-(1 →	→ 3,6)-Glc*p*-(1 →	→ 4,6)-Glc*p*- (1 →
YPG	10.9	32.0	37.3	12.2	0.7	1.8	1.8	3.4
YP_Glyc 10%	5.5	45.3	33.1	6.7	0.9	2.6	2.2	3.7
DPJW_Glyc 7.5%	5.8	49.7	28.5	6.5	0.5	3.0	2.8	3.3
DPJW_Glyc 10%	4.7	60.0	19.5	7.1	0.6	1.9	3.4	2.8
YP_Glyc 10% + Glc 1.5%	6.7	43.4	30.6	10.1	0.7	1.3	3.4	3.7
DPJW_Glyc 10% + Glc 1.5%	5.5	50.5	28.1	6.0	1.1	4.0	2.1	2.8
YP_Malt 10%	5.1	45.6	35.1	3.3	1.9	3.2	2.1	3.8
DPJW_Malt 10%	5.0	55.3	28.5	3.9	0.6	1.2	2.6	2.9

*YPG* yeast extract + pepton + 2% glucose medium, *YP* yeast extract + peptone, *DPJW* deproteinated potato juice water, *Glyc* glycerol, *Glc* glucose, *Gal* galactose, *Malt* maltose

Total branching ratio of studied glucans varies in approximate ratios from 1: 8 in glucan isolated from the biomass obtained in YP with maltose to 1: 12 in samples from DPJW with the addition of 7.5% of glycerol and DPJW with maltose (Table 4). Branching of 1,3-backbone to 1,6-sidechain occurs in the range of 1:12 in samples from YP with 10% glycerol and 1.5 glucose to 1: 27 in DPJW with the same combination of carbon sources.

The presence of 2,6-Me_2_ and 2,3-Me2 (3,4- and 4,6-branching points), together in approximate ratio from 1: 10 in glucan preparations after yeast cultivation in YP media with 10% of maltose, to 1: 21 in preparation isolated from biomass cultivated in DPJW with 10% of glycerol, indicates the binding between yeast glycogen and 1,3-backbone with 1,6-branches.

Various branching points suggest the complexity of glucan structure, also possible linking with the other cell wall components (e.g., polysaccharides) in the case of 4,6-Me_2_ (2,3-branching point) [42]. The values of relative mole % of → 4)-Glc*p*-(1 → type of linkage in isolated glucan preparations seems to be related to the lower susceptibility of the examined cells to the previously discussed *β*-(1,3)-glucan immunolabeling. At the same time the higher proportion of → 3)-Glc*p*-(1 → linkage facilitated interaction with the antibody (see results of *β*-glucan immunolabeling). This is also supported by the results presented in Table 5. Glucan samples from yeast cells in which greater susceptibility to immunolabelling was identified, were characterized by a higher glucan to glycogen ratio. At the same time, the conformation of the *β*-(1,3)-glucan molecules is very complicated, because the main chain tends to form helices. That is why it is difficult to draw one, clear conclusions why some of the studied cells were more prone to interact with the monoclonal-Abs used in our study.

**Table 5:** Results of glucan (range)–glycogen ratio estimation based on linkage analysis of studied glucan preparation depending on yeast cultivation media

Sample	SC	BF	Glucan (range)–glycogen ratio (estimate)
YPG	7.7	18	1.1–0.6: 1
YP_Glyc 10%	4.1	21	1.4–1.1: 1
DPJW_Glyc 7.5%	3.8	24	1.8–1.3: 1
DPJW_Glyc 10%	3.1	18	2.9–2.1: 1
YP_Glyc 10% + Glc 1.5%	3.9	13	1.6–1.0: 1
DPJW_Glyc 10% + Glc 1.5%	2.5	21	1.8–1.4: 1
YP_Malt 10%	2.6	22	1.2–1.1: 1
DPJW_Malt 10%	3.1	18	1.9–1.6: 1

*YPG* yeast extract + pepton + 2% glucose medium, *YP* yeast extract + peptone, *DPJW* deproteinated potato juice water, *Glyc* glycerol, *Glc* glucose, *Gal* galactose, *Malt* maltose

YPG – yeast extract + pepton + 2% glucose medium, YP – yeast extract + peptone, DPJW – deproteinated potato juice water, Glyc – glycerol, Glc – glucose, Malt – maltose

The analysis of individual peaks relative quantity, side-chain length (SC) and branching frequency (BF) parameters were evaluated by NMR using equations (SC = H6SC / (H6SC – H1SC)) and (BF = H1BB / (H6SC – H1SC). The estimation of relative quantity of *β*-(1,3)- and *β*-(1,6)-chains in glucan preparations were also evaluated based on NMR spectra analysis (Table 6). The higher number of BF means the higher gap, after which the next branching point occurs in *β*-glucan chain. Then, the lower the number of BF is the higher number of 1,6-branching is observed in glucan polymer. But the relative amount of 1,6-branching is also related to side chain length (SC). Therefore, the higher the SC is the higher content of 1,6-branching occurs in glucan structure. Obtained results confirmed differences in the structure of the *β*-(1,3/1,6)-glucan polymer. The studied glucan preparations differed in side-chain length and branching frequency, as well as in quantity of *β*-(1,3)- and *β*-(1,6)-chains. The biomolecules isolated from the biomasses grown in media containing maltose, regardless of the nitrogen source, were characterized by the lowest degree of *β*-(1,6)-branching. The presence of glucose at low concentration in media with glycerol as a dominant carbon source favored the process of *β*-(1,3)-glucan branching via *β*-(1,6)-chains, both in YP and DPJW media. For samples isolated after yeast cultivation in media supplemented only with glycerol, a greater degree of molecular branching was typical in media with YP as the nitrogen source.

**Table 6:** Individual peaks relative quantity, side-chain length (SC) and branching frequency (BF) parameters obtained from NMR using equations (SC = H6SC / (H6SC – H1SC)) and (BF = H1BB / (H6SC – H1SC) and estimation of relative quantity of *β*-(1,3)- and *β*-(1,6)-chains in glucan preparations isolated from chosen biomass after 48 h of cultivation

Sample	H6SC	H1SC	H1BB	SC	BF	H1SC/H6SC	*β*-1,3-/%	*β*-1,6-/%
YPG	133	101	1000	4.1	31	0.76	88	12
YP_Glyc 10%	226	198	1000	8.1	36	0.88	82	18
DPJW_Glyc 7.5%	177	136	1000	4.4	25	0.77	85	15
DPJW_Glyc 10%	148	114	1000	4.3	29	0.77	87	13
YP_Glyc 10% + Glc 1.5%	264	203	1000	4.4	17	0.77	79	21
DPJW_Glyc 10% + Glc 1.5%	188	140	1000	3.9	21	0.74	84	16
YP_Malt 10%	76.9	62.7	1000	5.4	71	0.82	93	7.1
DPJW_Malt 10%	88.8	58.9	1000	3.0	33	0.66	92	8.2

*YPG* yeast extract + pepton + 2% glucose medium, *YP* yeast extract + peptone, *DPJW* deproteinated potato juice water, *Glyc* glycerol, *Glc* glucose, *Gal* galactose, *Malt* maltose

As it was described in methodology section, also the solubility of studied glucans in 2.5 mass % LiCl/DMSO-d6 differed. In some cases, highly viscose or opaque gel was formed, but no clear correlation between solubility and SC/BF could be estimated. At the same time, clear relationship between the observed susceptibility of *Candida utilis* cells from indicated growth conditions to immunolocalization of *β*-(1,3)-glucan and the side-chain length and branching frequency in studied polymer is not possible to be addressed. Therefore, there were more factors that influenced the process, possibly other biomass components (e.g., *α*-glucan). The ^1^H NMR spectra indicated that all glucan preparations were contaminated with varying amounts of glycogen (≈ 5.0 ppm) and probably lipids (≈ 1.2 ppm and ≈ 0.8 ppm)—Table 7). From spectra of the analyzed glucans, it was apparent that the main portion of the glycogen was 1,4-linked Glc, there was no or hardlya trace of the peaks of 1,6-linked Glc (around 4.7 ppm).

**Table 7:** Glycogen content estimation in the glucan (glucan–glycogen complex) based on ^1^H NMR analysis

Sample	H1SC	H1BB	H1gly	Glycogen/%	Glucan/%	Glucan–glycogen ratio
YPG	101	1000	260	19	81	4.2:1
YP_Glyc 10%	198	1000	718	37	63	1.7:1
DPJW_Glyc 7.5%	136	1000	529	32	68	2.1:1
DPJW_Glyc 10%	114	1000	292	21	79	3.8:1
YP_Glyc 10% + Glc 1.5%	203	1000	608	34	66	2.0:1
DPJW_Glyc 10% + Glc 1.5%	140	1000	442	28	72	2.6:1
YP_Malt 10%	63	1000	496	32	68	2.1:1
DPJW_Malt 10%	59	1000	464	30	70	2.3:1

*YPG* yeast extract + pepton + 2% glucose medium, *YP* yeast extract + peptone, *DPJW* deproteinated potato juice water, *Glyc* glycerol, *Glc* glucose, *Gal* galactose, *Malt* maltose

The animal intestinal environment is constantly screened and processed by the diverse immune cells of the gut-associated lymphoid tissue because constant exposition to self, commensal and potentially pathogenic antigens exist. Therefore, the interaction between mucosa-associated lymphoid tissues and functional feed additives and influence of this interaction on animal health should be understood [14, 17]. Bastos et al. [14] indicated that brewery’s spent yeast glucan network containing *α*-(1,4)-, *β*-(1,4)-, and *β*-(1,3)-linkages allow to pose new research questions considering the recognition of the macromolecules-complex by key cell immune receptors. They underlined that beyond the recognition by immune-receptors of *β*-(1,3)-glucans, promoting yeast microcapsules internalization, the *α*-(1,4)-glucans can also interact with key receptors like Dectin-1 and DC-SIGN, acting as immune modulators. The DC-SIGN present in dendritic cells and macrophages recognizes glucans with preference for *α*-(1,4)-Glc, including also *β*-(1,4)-Glc linkage. Yeast may activate in vivo different pathways for stress adaptation, including architectural strategies altering cell surfaces to mask epitopes of innate immune pattern recognition receptors or enhance immune recognition and responses [33]. It was noticed that one type of glucan, *α*-(1,3)-glucan, present in filamentous fungi and dimorphic yeast, blocks the innate immune recognition by the *β*-glucan receptor [45]. It is by masking the last polysaccharide on the cell wall surface. The recognition by the innate immune system may be increased by defects in cell wall integrity [46]. It is worth determining how receptors of different immune cells respond to the presence of complexes of various types of glucan polymers in yeast cell wall. Besides, in vivo competition experiments between pathogenic and commensal *Candida* sp. have evidenced correlation between cell wall *β*-glucan exposure and the ability of commensal strains to colonize the mammalian gastrointestinal track in mouse model, suggesting that yeast cell wall architecture is crucial for colonization success [47]. Carrying out similar research would help to understand the properties of the obtained yeast biomass from applied breeding conditions in the context of its potential ability to modulate animal immune system.

## Conclusions

The results of the study showed that the composition of studied yeast biomass, considering especially the content of *β*-(1,3/1,6)-glucan, *α*-glucan and trehalose differed depending on growth medium of studied yeast. The highest *β*-(1,3/1,6)-glucan content and yield were observed after cultivation in deproteinated potato juice water as a nitrogen source and glycerol as a carbon source. The differences in the susceptibility of *β*-glucan localized in cells to interaction with specific *β*-(1,3)-glucan monoclonal antibodies was noted depending on the culture conditions. The polymer in cells from the DPJW and glycerol and galactose as a carbon source were labelled with the antibody with highest intensity. Obtained results confirmed differences in the structure of the *β*-(1,3/1,6)-glucan polymers considering side-chain length and branching frequency, as well as in quantity of *β*-(1,3)- and *β*-(1,6)-chains, however, no visible relationship was observed between the structural characteristics of the isolated polymers and its susceptibility to immunolabeling in whole cells. Presumably, other cellular components could have a masking effect on these interactions. *β*-(1,3)-Glucan was more intensely recognized by monoclonal antibody in cells with lower trehalose and glycogen content. This suggests the need to cultivate yeast biomass under appropriate conditions to fulfil possible therapeutic functions, however our in vitro findings should be confirmed in further studies using tissue or animal models.

### Supplementary Information

**Additional file 1: Table S1.** FTIR-ATR spectra of isolated *β*-glucan preparations.

## Data Availability

There is not any research data outside the submitted manuscript file.
