# Supplementary figures and images for: How do the carbon and nitrogen sources affect the synthesis of β-(1,3/1,6)-glucan, its structure and the susceptibility of Candida utilis yeast cells to immunolabelling with β-(1,3)-glucan monoclonal antibodies?
Source: Microb Cell Fact. 2024 Jan 19;23:28. doi: 10.1186/s12934-024-02305-4 (PMC10799355; doi:10.1186/s12934-024-02305-4)

**ADDITIONAL MATERIAL**

Table S1. FTIR-ATR spectra of isolated *β*-glucan preparations.


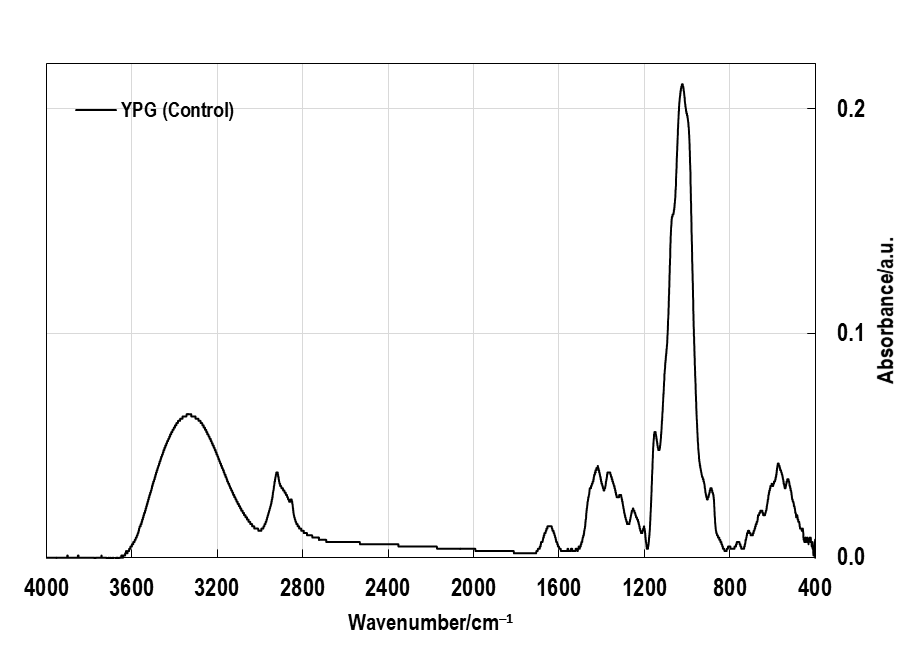

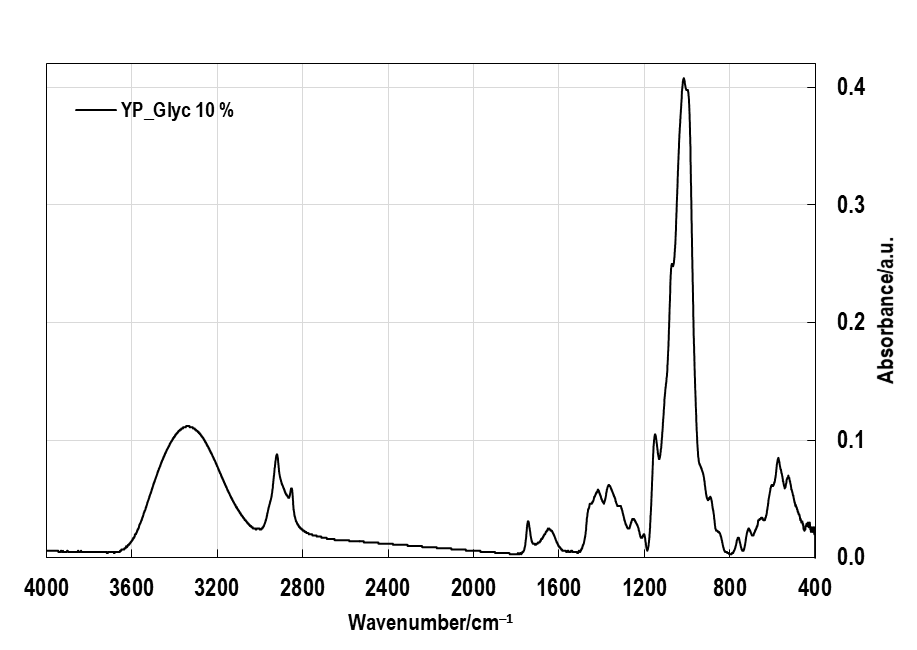


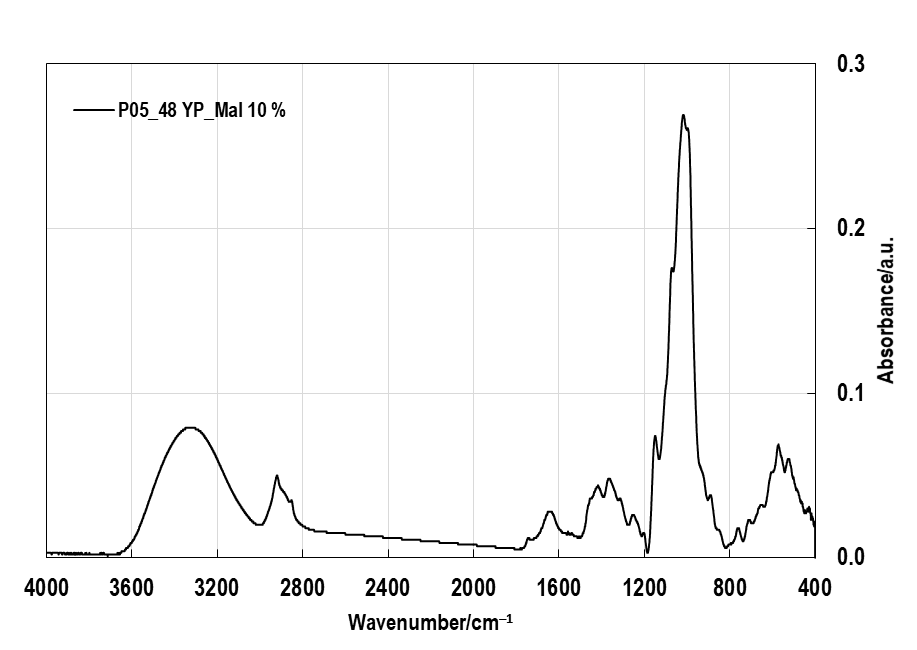

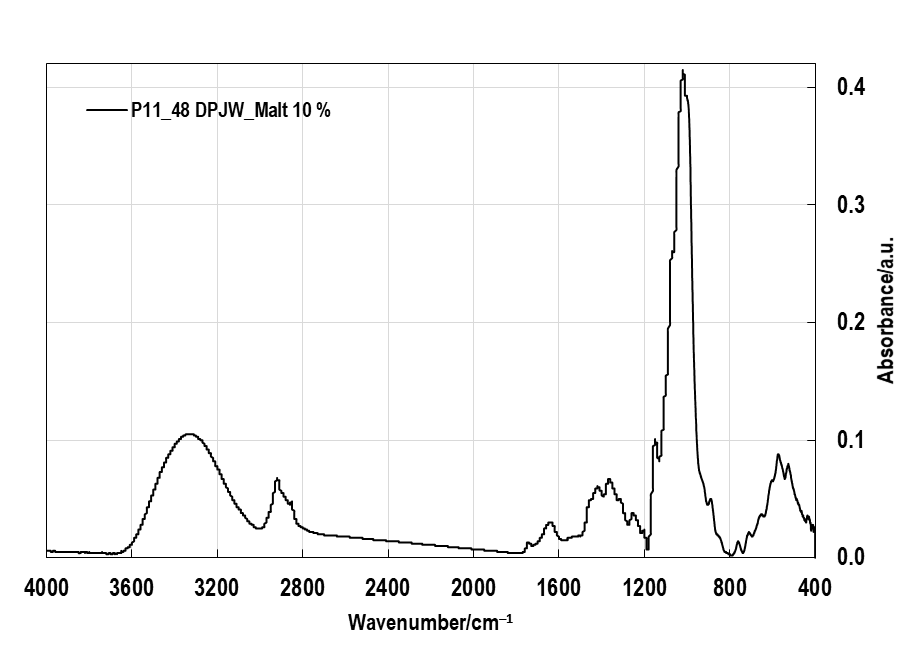


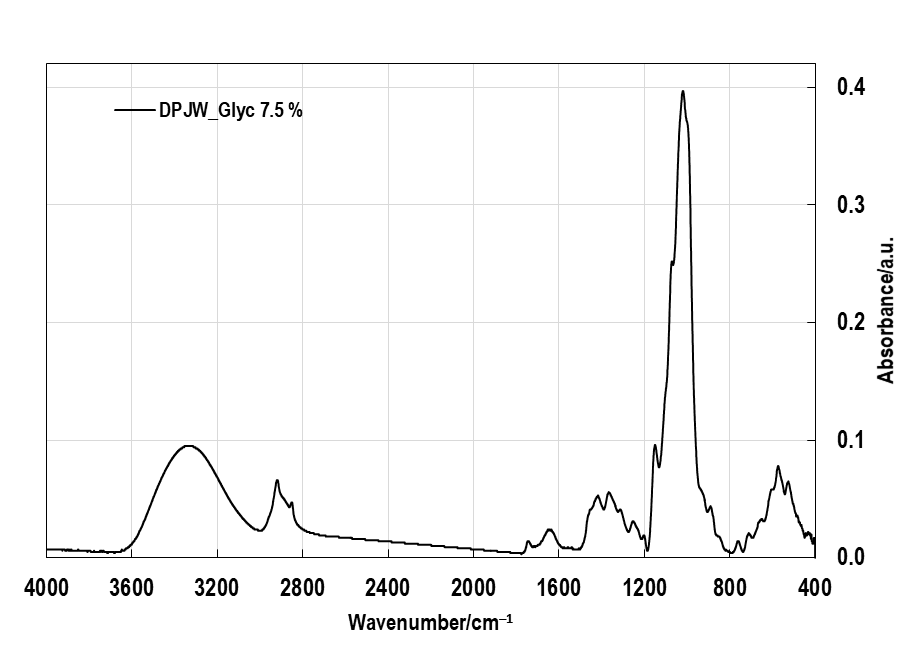

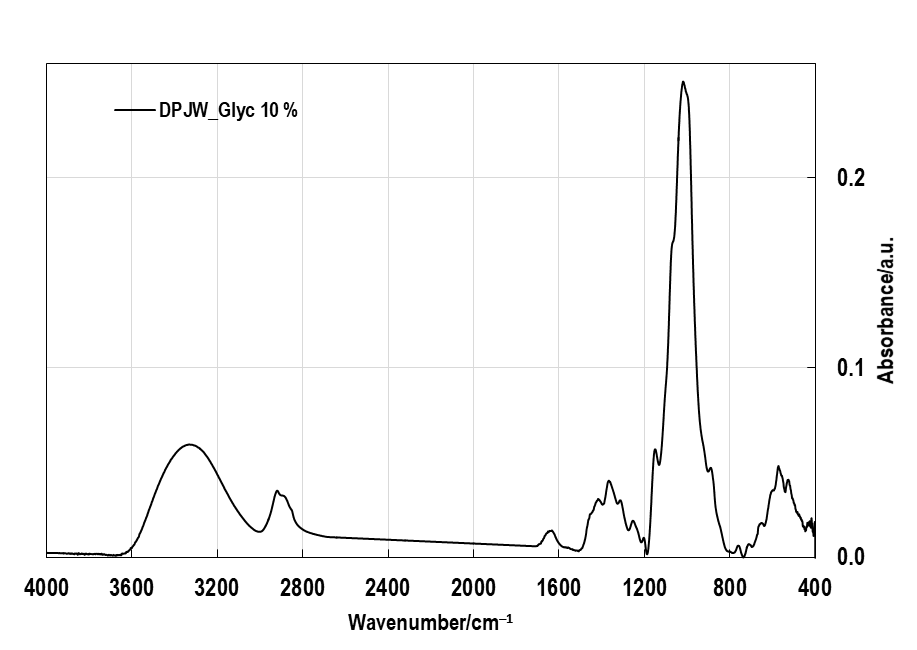


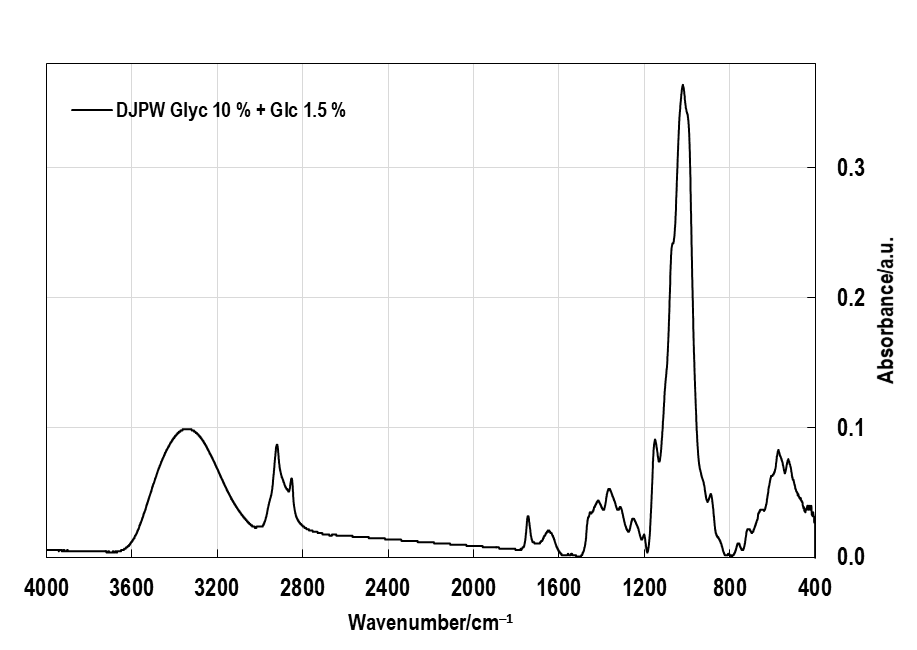

Supplement: Supplementary file 1 — Additional file 1: Table S1. FTIR-ATR spectra of isolated β-glucan preparations. [file 12934_2024_2305_MOESM1_ESM.docx]
